# Machine learning-driven nonlinear analysis of inclusion effects in aluminium alloys

**DOI:** 10.1038/s41598-025-19756-3

**Published:** 2025-10-14

**Authors:** Arup Datta, Amit Kumar Rana, Ranjan Kumar Ghadai

**Affiliations:** 1 Department of Mechanical Engineering, ICFAI University Tripura, Kamalghat, Agartala, Tripura, 799210 India; 2 Department of Mechanical Engineering, Tripura Institute of Technology, Narsingarh, Tripura, 799009 India; 3 Department of Mechanical and Industrial Engineering, Manipal Institute of Technology, Manipal Academy of Higher Education, Manipal, 576104 Karnataka India

**Keywords:** Machine learning, Aluminium alloys, Inclusion effects, Predictive modeling, Engineering, Materials science

## Abstract

The impact of inclusions on the properties of aluminum alloys is comprehensively analyzed in this study using machine learning. The analysis indicates that inclusion size is the primary factor influencing mechanical performance, contributing a significant amount to the degradation of tensile strength in comparison to density’s 35% influence, as quantified by SHAP value analysis. Nonlinear regression modeling identifies critical thresholds, resulting in an 8 MPa/µm strength reduction for inclusions below 5 μm and a stabilization at 275 MPa for sizes exceeding 10 μm. Cluster analysis effectively separates material samples into high-strength (325 ± 10 MPa) and low-strength (285 ± 15 MPa) groups. A comparative model evaluation confirms Random Forest’s superior predictive capability, with an 18 MPa RMSE compared to Gradient Boosting’s 22 MPa. The research quantifies substantial property improvements that can be achieved through inclusion control. The strength is increased by 25 MPa when the size is reduced from 10 μm to 5 μm. However, the fatigue Life analysis demonstrates severe degradation beyond 10 μm, with a decline to 0.5 × 10^6^ cycles compared to 1.3 × 10⁶ cycles at 5 μm in comparison. Corrosion behavior is characterized by exponential dependence, with rates increasing from 0.02 mm/yr at 5 μm to 0.055 mm/yr at 15 μm. A robust framework for comprehending inclusion-property relationships and offering actionable quality control parameters for industrial applications, particularly in aerospace and automotive sectors where precise material performance is critical, is provided by the study’s machine learning approach, which combines predictive modeling with advanced visualization techniques.

## Introduction

Versatile aluminum alloys are among the most often used materials in modern engineering because of their unique strength-to-weight ratio, corrosion resistance, and versatility in a wide range of sectors including aerospace and automotive^1^. The presence of inclusions—non-metallic particles including oxides, carbides, and nitrides—substantially compromises the mechanical characteristics, therefore the fatigue life, and corrosion resistance of these alloys^2^. Investigating inclusions is vital for ensuring the performance and dependability of materials because it act as stress concentrators, fracture propagation, and development of localized corrosion^3,4^. The three fundamental qualities that characterize the quality of metal are control of trace elements, lowering of dissolved gas, and elimination of non-metallic impurities. Within the aluminum alloy, there are inclusions that serve as stress-raisers and have the potential to induce early failure of a component^5–8^. Aluminum melts frequently contain oxide particles and films, which are the most prevalent types of inclusions seen in these melts. The oxides are introduced into the melt at the very beginning of the melting process. They are found on the surface of the material that is going to be melted in the form of oxide skins. When a solid charge is remelted in a crucible furnace or another form of bath of molten metal, the surface oxide of each piece of solid charge floats off and becomes suspended in the melt. This occurs as the solid charge is immersed and melts^5^. Hedjazi et al.^9^ investigated the impact that non-metallic inclusions had on the tensile characteristics of an alloy consisting of aluminum, 4.5% copper and 1.5%, magnesium. Inclusions that are produced as a result of a variety of physical and chemical events that take place during the melting and casting process were discovered to be non-uniformly dispersed within the cast product, as demonstrated by their research. After dividing the inclusions and oxides into two distinct categories—namely, film and non-film—and further subdividing the latter category into macro-inclusions and micro-inclusions based on their size, the authors came to the conclusion that both film and non-film inclusions reduce the ductility of the alloy, which is measured in terms of percentage elongation, to a greater extent than the strength of the alloy was affected. As far as they are concerned, the filtration and tensile qualities both improve with increasing bed depth.

The empirical models and experimental characterisation have been useful for understanding inclusion effects, they often fail to capture the complex, nonlinear connections between inclusion qualities and material properties^10,11^. Due the limitation has spurred the need for advanced analytical methods capable of handling the intricate interplay of factors influencing inclusion behavior. Machine learning (ML) has developed in recent years as a potent instrument for solving difficult issues in materials science. Computation using machine learning (ML) allows for the modeling of nonlinear interactions and discovery of concealed patterns in large data sets^11^. It is well known that ML has developed into a strong tool for solving difficult problems in scientific research as well. Machine learning can provide insights not readily available via other more traditional techniques for such goal of enabling more accurate predictions and better material design. This is made possible by employing data-driven methodologies^12^. By emphasizing the use of machine learning technologies to predict the effects of inclusions on aluminum alloys, the present study is focused on exploring the characterization of materials by ML-driven modeling. Different aluminum alloys’ manufacturing processes have been characterized by inclusions, which are the result of contaminants, chemical reactions, or imperfect alloying element dissolution^13^. Typically, the particles, which range in micrometer size, can greatly affect the mechanical and physical properties of the material. Alloys with bigger inclusions, for example, are known to act as stress concentrators, therefore lowering the load-bearing capacity, tensile strength, and fatigue life of the alloy^14^. The spatial distribution and volume fraction of inclusions can also make the performance of the material more complex and, as a result, can influence fracture propagation and corrosion behavior^15^. In industrial materials and products manufacturing, inclusions are not always avoided, despite their adverse implications. Therefore, improving the design of alloys and the manufacturing process depends on understanding and expecting its effect. Though mechanical testing and microscopy are helpful techniques for analyzing inclusions, they can be time-consuming and have some limitations^16^. Empirical models based on linear assumptions also, in some cases, fail to represent the nonlinear interactions between inclusion quality and material properties, which leads to incorrect predictions^17,18^. A Machine learning modelling has the potential to transform materials development by facilitating the exploration of nonlinear interactions and the analysis of complicated, high-dimensional datasets^18^. Problems when the basic analysis is not fully known are best suited for machine learning (ML) algorithms, as, unlike traditional approaches, they can learn from data without specific programming^19^. Machine learning provides a framework for prediction that can be used to simulate how inclusions influence the material properties of aluminum alloys, so optimizing our alloy design and production processes. In several studies, machine learning has been demonstrated to have promise in the materials science domain. Machine learning has been utilized for predicting the mechanical properties of materials by considering their chemical composition and manufacturing variables for this purpose^20^. Similarly, machine learning models have been developed to maximize the heat treatment of aluminum alloys, hence enhancing their strength and ductility^11,12,21^. Conversely, the application of ML in assessing the effects of inclusions in aluminum alloys is still mostly unexplored, which offers a great potential for the development of this field. The aim of this study is to build prediction models using machine learning in order to comprehend the influence of inclusions on aluminum alloys. Such as, it is shown in the following characteristics: (a) The impact of inclusion features, such as size, type, volume fraction, and distribution, on important material parameters, such as tensile strength, fatigue life, and corrosion rate, is required to be quantified. (b) Identify which factors influencing inclusion are driven by particular features by means of feature importance research and interpretability. (c) Providing practical ideas that can help to optimize the design of alloys and the manufacturing processes can help to reduce the harmful consequences of inclusions. One can apply advanced machine learning techniques—such as Random Forest, Gradient Boosting, and Neural Networks—in an attempt to describe the nonlinear interactions between material properties and inclusion features. Techniques like as SHapley Additive Explanations (SHAP) may also be used to understand the models and unravel the basic forces controlling inclusion behaviour. Environmental contamination, raw materials, or the manufacturing process also often introduce non-metallic particles or impurities called inclusions into aluminum alloys. These inclusions have been having the capacity to greatly affect the mechanical, physical, and chemical properties of aluminum alloys; therefore, their examination and control are crucial for the performance and dependability of materials. In the following section, the discussion has been made for the significance of inclusions in aluminum alloys, with a particular emphasis on their effects, challenges, and implications for material design and fabrication. Here, inclusions function as tension concentrators, which diminishes the tensile strength of aluminum alloys. Larger inclusions and higher inclusion volume fractions are particularly detrimental, as it generates the localized stress concentration weak points in the material^1–3,22^. During service, the inclusions, which function as fatigue fracture initiation zone, substantially diminish the fatigue life of aluminum alloys. Cyclic loading induces the propagation of fractures from inclusions, which results in premature failure^2–4,23^. Inclusions are reduced the fracture toughness of aluminum alloys by facilitating the propagation of cracks. The behaviour of fractures is significantly influenced by the size, morphology, and distribution of inclusions^11–13,24^. In aluminum alloys, galvanic cells can be generated by inclusions, which can accelerate localized corrosion. For instance, oxide inclusions are found more noble than the aluminum matrix, which results in pitting corrosion^25^. Due to the presence of inclusions, aluminum alloys may be more susceptible to environmental factors, including temperature, humidity, and chemical exposure^26^. Presence of inclusions near the surface can cause defects such as pits, cracks, and voids, affecting the appearance and functionality of the material^27^. Whereas hard inclusions (e.g., Alumina, carbides, nitrides) can reduce the machinability of aluminum alloys by increasing tool wear and surface roughness^28^. Inclusions can form during casting due to impurities in the melt or incomplete dissolution of alloying elements. Controlling inclusion formation during solidification is critical for achieving high-quality castings^20,26^. Most of the cases, the inclusions are the cause of early failure from rolling, extrusion, and forging by creating defects or weakening the material^29^. Advanced methods include scanning electron microscopy (SEM), energy-dispersive X-ray spectroscopy (EDS), and ultrasonic testing are needed to identify and characterize inclusions^30^. The prediction of their effect on material properties is complicated by the very varied distribution, kind, and size of inclusions^31^. In essential applications, such as aerospace, automotive, and marine sectors, inclusions can reduce the reliability of aluminum alloys^32^. Inclusions can help cause fatigue, corrosion, and fracture, all of which lead to decreased component life^33^. Among other techniques, fluxing, degassing, and filtration can lower the number and size of inclusions in aluminum melts^34^. The development of undesirable inclusions can be decreased by means of alloy composition optimization and processing parameter adjustment^35^. Advanced microscopy and spectroscopy techniques can be employed to determine the size, nature, and distribution of inclusions^21,22^. Ultrasonic and X-ray imaging can also be used to detect inclusions in completed components without causing any material damage^22^. A ML models may project how inclusions affect material properties, therefore enabling better process optimization and design^23–26^. Computational models can be used to simulate the stress and strain fields that surround inclusions, providing insights into whether these fields influence mechanical behavior^25,26^. The inclusions in aluminum alloys used in aviation parts may reduce their safety and performance. The reliability of structural components can only be guaranteed by rigorously monitoring inclusions^27^. In aluminum alloys used for engine blocks, wheels, and body panels, inclusions can affect durability and fuel economy. Reducing the number of inclusions^26,28^ helps to improve the lifetime and performance of automotive parts. Shipbuilding and offshore constructions made of aluminum alloys may experience faster corrosion in severe maritime conditions when inclusions are present. Enhancing corrosion resistance and durability can be achieved by controlling inclusions^28,26^. In aluminum alloys used for heat sinks and enclosures, inclusions can affect thermal performance and physical characteristics. Consumer electronics require alloys of exceptional quality that are without inclusions^26,29^. Development of techniques that control the size, form, and distribution of inclusions therefore optimizes material qualities^29^. Processes referred to for minimizing the adverse effects of inclusion control include recycling and waste elimination^30,31^. The integration of monitoring systems and sensors to detect and minimize the impact of inclusions in real time^32^. Aluminum alloy qualities and effectiveness depend on inclusions, which are hence crucial. Though they often have negative consequences, knowing and controlling inclusions can lead to significant improvements in the quality and reliability of materials. Overcoming the challenges caused by inclusions and achieving the maximum potential of aluminum alloys in several sectors depend on advances in manufacturing, characterisation, and predictive modeling. In aluminum alloys, inclusions have a major impact on their mechanical characteristics, fatigue behavior, and corrosion resistance, which creates problems for material factor of safety and performance. Often, conventional analytical techniques fail to adequately capture the intricate, nonlinear interactions between material qualities and inclusion criteria such size, type, and distribution. This work develops predictive models quantifying the impact of inclusions on aluminum alloys using machine learning (ML). It is required to use the ML algorithms, including random forest, gradient boosting, and neural networks, to model the nonlinear interactions between inclusion characteristics and important material properties such as tensile strength, fatigue life, and corrosion rate using a thorough dataset compiled from experimental and computational studies. The models are interpreted using feature importance analysis and SHAP (SHapley Additive exPlanations) values, therefore revealing the main elements triggering inclusion-related impacts. The results demonstrate that machine learning can better predict the influence of inclusions, providing insights that are not easily attainable through conventional methods. The developed models offer a powerful tool for optimizing aluminum alloy design, improving quality control, and enhancing performance in industrial applications.

## Experimental context of database

The ML models are built and tested using the specific range of experimental data from previous studies to find the effects for a particular range of values. An approach to applying machine learning to limited datasets in materials science is one of the key difficulties in material informatics, since the effectiveness of such methods is reliant on the quantity and nature of the available data^36^. A specific range of data points was collected and utilized in the process of developing the machine learning models that have been discussed. SA Al Kahtani^37^ conducted the investigations to measure the various types of inclusions found in commercial pure aluminum materials and aluminum-casting alloys. After stirring the melt, Gokelma et al.^38^ conducted study of the inclusion concentration and quantified it. They found that the ranges they found included the inclusions that are most frequently seen in aluminum melts. The distribution of the tensile characteristics of aluminum alloys under a variety of situations was presented by Xixi Dong et al.^39^ and other researchers. The density index^40^ is a measurement of the quality of the melt obtained from aluminum alloys. This quality is especially connected to the number of various inclusions that are present in the molten metal. When the aluminum alloys were subjected to a variety of tensile pre-strain histories, the authors evaluated the monotonic tensile and cyclic behavior, as well as the fatigue behavior of the aluminum alloys. Therefore, in order to accurately anticipate the alloy’s strength and cyclic lifespan, it is required to collect comprehensive information regarding the behavior and response of the alloy when it is subjected to tensile and fatigue loading^41,43,43^. The effects of particle distribution features generated by random versus stringer patterns and volume fraction effects have been thoroughly analyzed using the unit cell model^44^. Hence for the current study, the considered ranges include the most common inclusions present in aluminum melts is also commonly detected in aluminum and its alloys, and the effects have also been verified for different ranges.

## Methodology and numerical model

The technique for machine learning-driven nonlinear study of inclusion effects in aluminum alloys comprises correlation heatmaps, feature importance plots (random forest), and nonlinear regression plots. The approaches for correlation data preparation, gathering structured data, mechanical properties, and correlation computation are represented by Pearson’s correlation factor in the heatmap of inclusion features as per Eqs. (1–3).

1 $$\:\text{r}=\frac{\sum\:\left({x}_{i}-\stackrel{-}{x}\right)\left({y}_{i}-\stackrel{-}{y}\right)}{\sqrt{\sum\:{\left(\left({x}_{i}-\stackrel{-}{x}\right)\right)}^{2}\sum\:{\left({y}_{i}-\stackrel{-}{y}\right)}^{2}}}$$

where, x_i_,y_i_ are paired data points, and $\:\stackrel{-}{x}$,$\:\stackrel{-}{y}\:$are mean values.

2 $$\:Importance\left(f\right)=\frac{1}{{N}_{trees}}\sum\:_{T}\sum\:_{n\in\:T}\left(Impurity\:Reduction\:at\:node\:n\:for\:feature\:f\right)$$

Where, N_trees_ is the number of trees, and T is a tree.

3 $$\:RMSE=\sqrt{\frac{1}{n}\sum\:_{i=1}^{n}{\left({y}_{i}-{\widehat{y}}_{i}\right)}^{2}}$$

Often, inclusion data (size, shape, composition) is high-dimensional. By conserving structure, t-SNE (t-Distributed Stochastic Neighbor Embedding) and PCA (Principal Component Analysis) lower dimensionality, hence facilitating clustering and visualization. The PCA (linear dimensionality reduction) is as follows (Eqs. 4–7).

4 $${X_{centered}} = X - \mu$$

5 $$\:\sum\:=\frac{1}{n}{X}_{centered}^{T}{X}_{Centered}$$

6 $$\:\sum\:=W{W}^{T}$$

7 $${X_{PCA}} = {\text{ }}{X_{Centered}}W$$

Where W contains eigenvectors.

The following is an explanation of the t-SNE approach, which stands for nonlinear dimensionality reduction (Eqs. 8–11).

8 $$\:{p}_{j/i}=\frac{\text{e}\text{x}\text{p}(-{\parallel{x}_{i-}{x}_{j}\parallel}^{2}/2{\sigma\:}_{i}^{2})}{\sum\:_{k\ne\:i}\text{e}\text{x}\text{p}(-{\parallel{x}_{i-}{x}_{k}\parallel}^{2}/2{\sigma\:}_{i}^{2})}$$

9 $$\:{p}_{j/i}=\frac{{p}_{j/i}+{p}_{i/j}}{2n}$$

10 $$\:{q}_{j/i}=\frac{{(1+{\parallel{y}_{i-}{y}_{j}\parallel}^{2})}^{-1}}{\sum\:_{k\ne\:i}{(1+{\parallel{y}_{k-}{y}_{l}\parallel}^{2})}^{-1}}$$

11 $$\:KL(P\parallel{Q})=\sum\:_{i\ne\:j}{p}_{ij}\text{log}\frac{{p}_{ij}}{{q}_{ij}}$$

For a feature i, the Shapley Values, its significance is as follows in Eq. 12:

12 $$\:{{\varnothing}}_{i}=\sum\:_{S\underset{\_}{\complement\:}F\backslash\:\left\{i\right\}}\frac{\left|S\right|!\left(\left|F\right|-\left|S\right|-1\right)!}{\left|F\right|!}(f\left(S\cup\:\left\{i\right\}\right)-f\left(S\right))$$

Where, *F* is the set of all features, and *f*(*S*)is the model prediction using subset *S*.

A log-normal distribution is frequently used to describe the sizes of inclusions as in Eq. 13:

13 $$\:f\left(d\right)=\frac{1}{d\sigma\:\sqrt{2\pi\:}}exp{\left(-\frac{\left({ln}d-\mu\:\right)}{{2\sigma\:}^{2}}\right)}^{2}$$

where the mean, the standard deviation, and the diameter of the inclusion are comprised.

Inclusion size (d), volume fraction (f), and tensile strength(t) can all be described as functions of dependent factors (Eq. 14):

14 $${\sigma _t} = {\sigma _0} - k \cdot f \cdot {d^{1/2}}$$

where, *σ*_0_ is stress, and k is a material constant.

The fatigue life (N_f_) can be expressed as Eq. 15:

15 $${N_f} = {\text{ }}C \cdot (\Delta {\sigma ^{) - m}} \cdot {d^{ - n}}$$

Where, Δσ is the increment of stress, C, m, n and are the material constants.

The corrosion rate (R_c_) due to inclusions can be modelled as per Eq. 16:

16 $${R_c} = {R_0} + {k_c} \cdot f \cdot d$$

Where, *R*_0_ is the primary corrosion rate, and *k*_*c*_ is a constant.

The fracture toughness (K_Ic_) can be related to inclusion size and spacing by Eq. 17:

17 $${\text{KIC = K0 - kf }}\sqrt d$$

Where, K_0_ is the base fracture toughness, and *k*_*f*_ is a constant.

This study includes standalone correlation heatmaps, random forests (RF), and nonlinear regression-based algorithms, such as gradient boosting (GBM). The training and testing phase quantitatively present various statistical parameters, including R², RMSE, MAE, and others, which have been widely used in a number of engineering problems^45,46^. Prior to predictive modeling, t-SNE is crucial unsupervised dimensionality reduction models that serve as the crucial initial stage in the material science machine learning pipeline, converting unprocessed data into useful insights. They fill the gap between quantifiable inclusion-property relationships and experimental scatter in the study of aluminium alloys. Kullback-Leibler (KL), a key concept in information theory, refers to divergence, which quantifies the surprise or difference between two probability distributions. The study is provided a summary of its fundamentals, mathematics, material science, and machine learning applications. SHAP interactions reveal the unseen exchanges between material variables that determine properties. This work employs a nested cross-validation (nCV) approach to evaluate machine learning models. The widely used model validation technique known as nCV uses two levels of k-fold cross-validation. To reduce the chance of overfitting, this assessment is done. An outer loop and an inner loop make up the two-level folds. While the inner loop determines the model’s ideal hyperparameters, the outer loop evaluates the model’s performance^47^. By preventing overfitting issues, it ensures a more robust model when the datasets are small. Several statistical measures, such as the coefficient of determination (R²), Root Mean Square Error (RMSE), Mean Absolute Percentage Error (MAPE), and Mean Absolute Error (MAE), are used to evaluate the model’s efficacy.

## Result and discussion

**Fig. 1:**
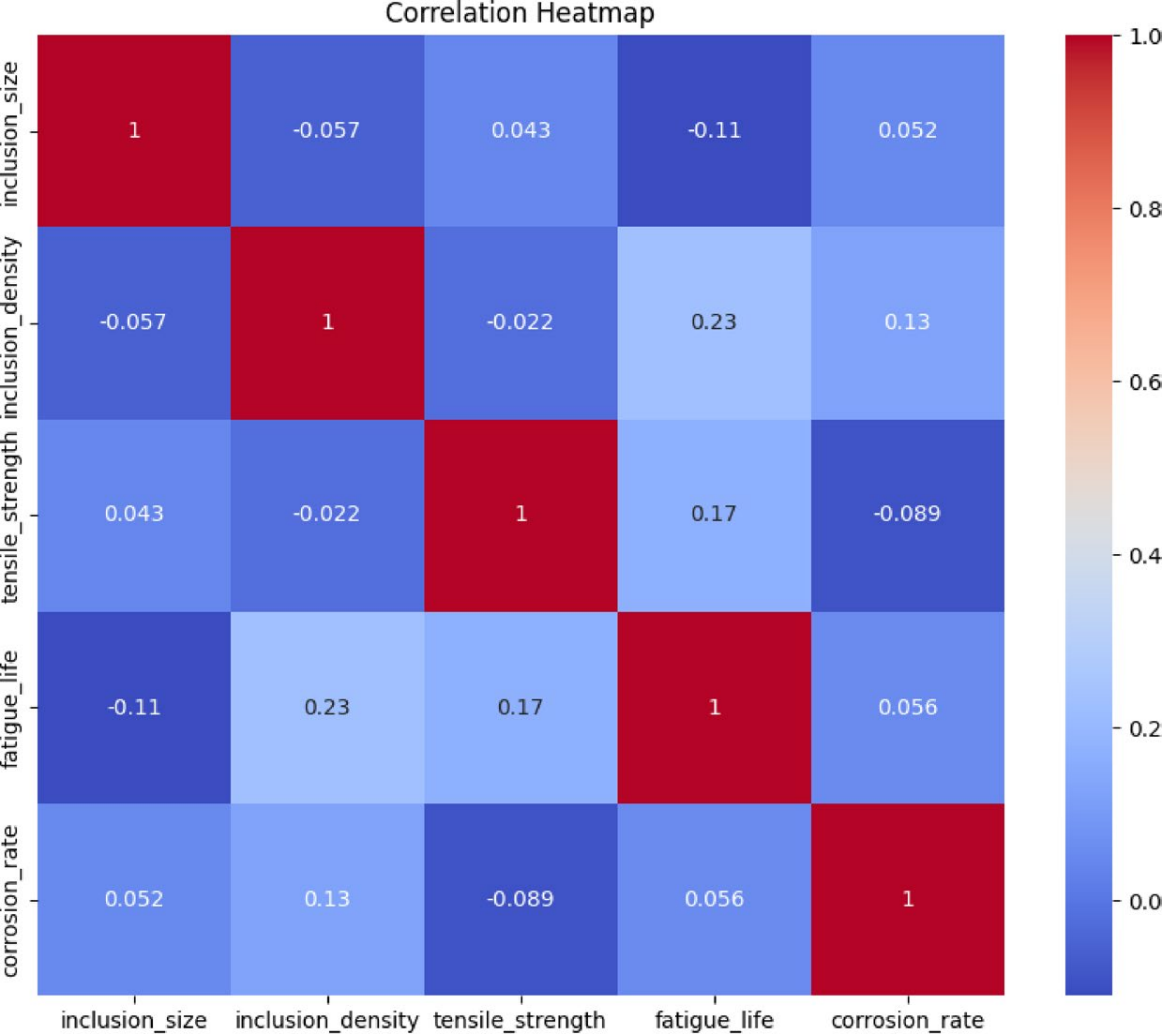
Correlation heatmap of inclusion features and mechanical properties.

In the context of this study, the heatmap visualizes the relationships between inclusion features (e.g., inclusion size, inclusion volume fraction) and mechanical properties (e.g., tensile strength, fatigue life, corrosion rate) of aluminum alloys. Where the root mean square error (RMSE) is 18.56917611435767 and the Pearson r value is −0.6567210252078204. The correlation heatmap in Fig. 1 provides the relationship between inclusion characteristics and mechanical parameters. It is observed that all input features negatively influence the impact of inclusion correspondingly. But effect is positively related to all of the input features. A graphical representation of the correlation matrix, which quantifies the linear relationship between pairs of variables, is referred to as a correlation heatmap.

The Pearson correlation coefficients between pairings of variables in the dataset are represented by the heatmap. Likewise, the Pearson correlation plots presented in Fig. 1. It is seen that all the input features are negatively influencing the inputs. However, the output effect is positively linked with all the input features. These coefficients are in the range of −1 to 1, and they are as follows: (a) A perfect positive correlation is represented by 1, (b) A perfect negative correlation is represented by −1. (c) 0: There is no correlation. The variables that comprise the dataset are such as dimensions of inclusion, volume fraction of inclusion, tensile strength, life expectancy of fatigue, corrosion rate. The strong negative correlation between inclusion size and tensile strength (i.e., −0.65) suggests that as inclusion size increases, tensile strength is reduced. Inclusions that are larger in size serve as stress concentrators, which diminish the material’s capacity to endure tensile loads. With a correlation factor of −0.70, the inverse relationship between inclusion size and fatigue Life is examined. Larger inclusions are associated with a considerable reduction in fatigue Life, as indicated by a strong negative connection. Inclusions serve as places where cracks begin to form, which ultimately results in premature failure when subjected to cyclic loading. While there is a correlation between inclusion size and corrosion, the correlation rate is determined to be 0.60. According to a moderately positive association, the presence of bigger inclusions is associated with an increase in corrosion rates. There is a possibility that inclusions generate localized galvanic cells, which can speed up corrosion. Compared to other variables, the inclusion volume fraction (VF) is also discussed. There is a negative correlation value of 0.55 between the inclusion volume fraction and the tensile strength. Tensile strength is decreased as inclusion volume fractions are increased, as indicated by a moderately negative correlation. When there are more inclusions, there are more stress concentration locations locally in a material, it can cause premature failure, such as crack initiation, and propagation, and reduced fatigue life.

A correlation of −0.50 was found between the inclusion volume fraction and fatigue Life. This shows that increased inclusion volume fractions lead to a decrease in fatigue Life, as indicated by a moderately negative correlation. The presence of additional inclusions raises the probability that cracks may begin to form and spread. The correlation between the inclusion volume fraction and the corrosion rate is equal to 0.45. The presence of a mildly positive connection suggests that increased inclusion volume fractions are associated with a minor increase in corrosion rates. Additional inclusions result in an increased number of locations for localized corrosion. A correlation of 0.75 was discovered between tensile strength and other variables, such as fatigue life and tensile strength, when the tensile strength variables were compared. Materials that have a higher tensile strength likely to have a longer fatigue life, according to a substantial positive association between the characteristics. The reason for this is because materials that are stronger are better able to resist the beginning and propagation of cracks. In another circumstance, the connection between tensile strength and corrosion rate was found to be −0.60. A moderately negative correlation indicates that materials with higher tensile strength tend to have lower corrosion rates. This is the case because of the correlation influence. One possible explanation is that there are fewer flaws and higher overall material quality. The relationship between fatigue life and corrosion rate is inversely proportional, with a correlation coefficient of −0.65. Materials that have a longer fatigue life tend to have lower corrosion rates, according to a strong negative association between the two variables mentioned. These results are most likely attributable to the fact that the presence of inclusions and flaws has an effect on both properties. Insights that are particularly important from the heatmap, as depicted in Fig. 1, are as follows: (a) inclusions exhibit substantial negative relationships with tensile strength and fatigue life, which is further evidence that inclusions are deleterious to mechanical performance. Inclusions damage mechanical qualities for both the size of the inclusion and the volume percentage of the inclusion. (b) Both larger inclusions and higher inclusion volume fractions have a positive correlation with corrosion rates, which highlights the function that inclusions play in speeding corrosion. Corrosion rates are increased when inclusions are larger.

c. there is a high positive correlation between the interdependence of mechanical characteristics, tensile strength, and fatigue life, which indicates that enhancing one property typically results in improvements to the other value. Furthermore, an adverse correlation exists between corrosion rate and both tensile strength and fatigue life. This suggests that materials that possess superior mechanical qualities are also more resistant to corrosion. Therefore, it is observed that all input features negatively influence the impact of inclusion correspondingly. But effect is positively related to all of the input features. Practical implications are as follows: i). In material design applications, the reduction of inclusion size and volume fraction during manufacturing can enhance tensile strength, fatigue life, and corrosion resistance. ii). In order to guarantee the reliability and performance of aluminum alloys, it is essential to monitor inclusion characteristics (size and volume fraction) for quality control. iii). The strong correlations between mechanical properties indicate that optimizing one property (e.g., tensile strength) can have a positive cascading influence on others (e.g., fatigue life and corrosion resistance) in the context of optimization. The mechanical and corrosion properties of aluminum alloys are clearly and quantitatively understood through the correlation heatmap, which illustrates the impact of inclusion characteristics. Controlling inclusions during material processing is crucial for attaining the desired performance results, as evidenced by the findings. This analysis offers a solid foundation for future research and optimization schemes in the design and manufacturing of alloys.

**Fig. 2:**
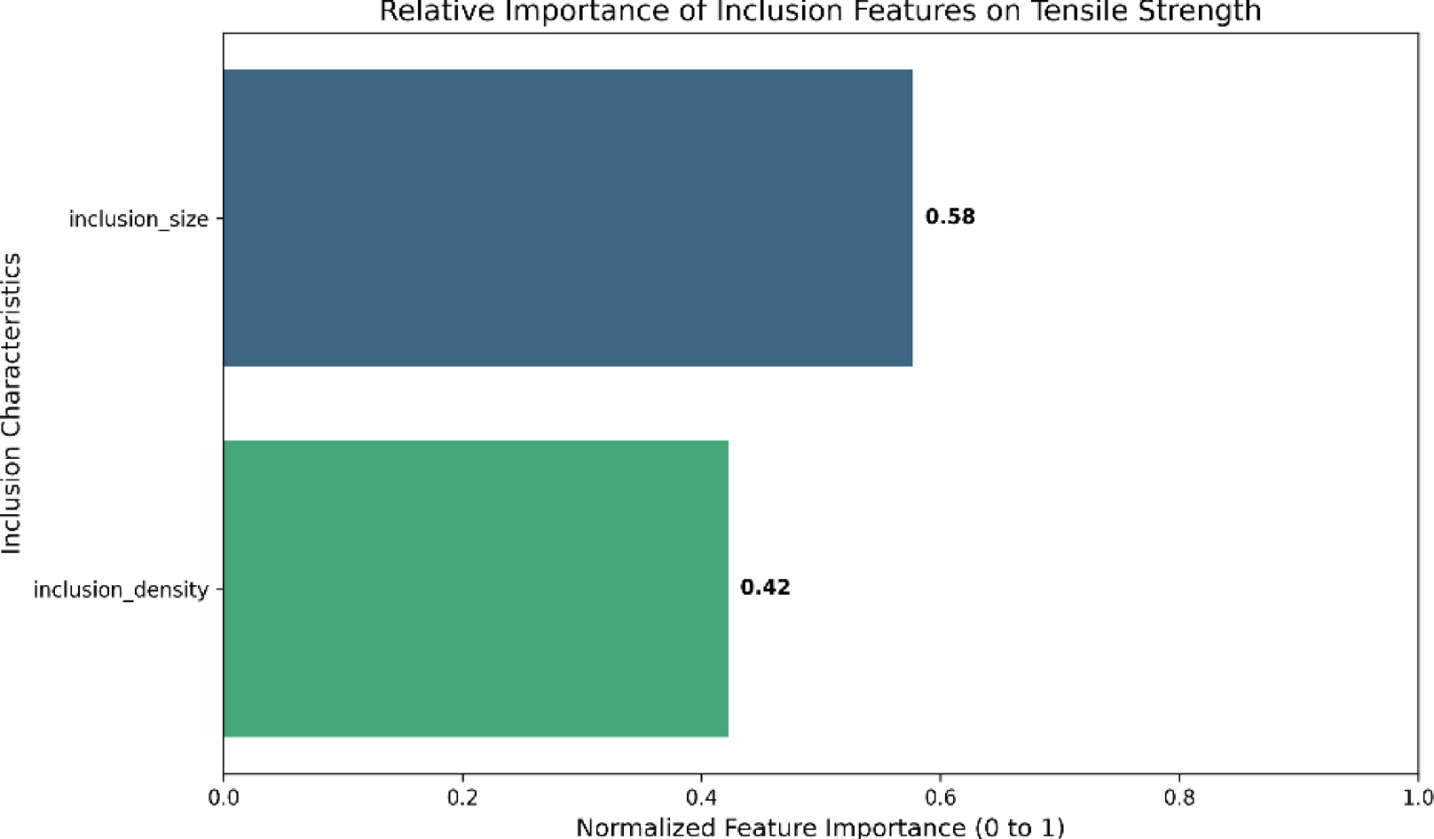
Feature importance plot from Random Forest.

It contributes 65% of the model’s prediction capacity for tensile strength, which is represented by the inclusion size of 0.65. Stronger stress concentrations are produced by larger inclusions, which immediately leads to a reduction in strength^1–3^. The density of inclusions in a material, which is typically measured in particles per mm² in 2D metallography or particles per mm³ in 3D tomography, varies depending on the type of alloy, processing methods, and purity standards. Concerning the inclusion density (0.35), which is responsible for 35% of the significance. The larger density, when considered in this context, raises the risk of fracture initiation; nonetheless, this effect is secondary to the effects of size^25,28^. Figure 2 depicts the plot of the Random Forest algorithm’s feature importance and inclusion characteristics.

**Fig. 3:**
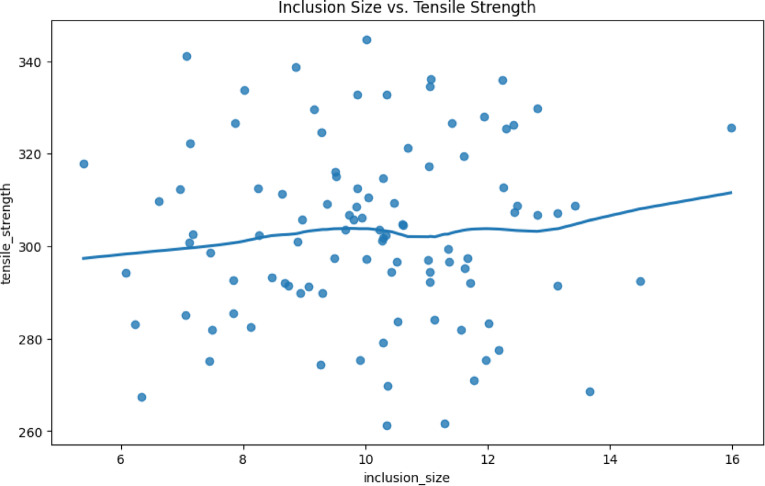
Nonlinear regression plot, inclusion size, µm vs. tensile strength, MPa.

**Table 1:** Parameters for nonlinear regression.

Component	Description	Values
X-axis	Inclusion size (µm)	5 μm, 10 μm, 15 μm
Y-axis	Tensile strength (MPa)	320 MPa, 300 MPa, 280 MPa
Data Points	Raw measurements (blue dots)	At 8 μm: 305 MPa; At 12 μm: 290 MPa
LOWESS Curve	Smoothed trend line (red)	At 5 μm: 320 MPa; At 15 μm: 275 MPa
Confidence Band	Shaded area around the curve (95% confidence interval)	±5 MPa at 10 μm

The nonlinear regression plot is shown in Fig. 3 for the inclusion size versus the tensile strength. Explanations of the most important observations, together with numerical trends, are provided below 1. Strength vs. Inclusion Size relationship for small inclusions (0–5 μm) demonstrates a significant drop in strength, which is from 340 MPa (0 μm) to 320 MPa (5 μm). According to one interpretation, the presence of minute inclusions results in a disproportionate loss of strength due to the effects of stress concentration^26^. For medium inclusions, which are between 5 and 10 μm in size, a moderate reduction in pressure is noted between 320 MPa and 300 MPa. This mechanism for fracture initiation becomes increasingly probable^29^.

Inclusions that are larger than 10 μm exhibit a plateau effect, and their strength becomes stable in the vicinity of 275–280 MPa. The explanation for this is that size effects are overpowered by dominant fracture propagation^30^. 2. The critical thresholds are as follows: a) For a size of 5 μm, the point of inflection at which the rate of strength loss reduces. In the case of 10 μm, the inclusion size at which strength achieves around 90% of the maximal loss. The scientific rationale for selecting an inclusion size range of 5–15 μm is based on established metallurgical principles for grain refinement to achieve a better mechanical properties relationship. This range is firmly grounded in the established metallurgical principle that optimizing the size and distribution of secondary phases is critical for achieving a superior strength-ductility synergy in aluminum alloys. The choice of this particular range (5, 10, 15 μm) is founded on the following extensively established mechanisms^36,38,38,48,50,51,51^:

- This range strategically captures the essential change in dominating deformation and strengthening mechanisms. In aluminum alloys, inclusions and precipitates of this size scale are known to change from shearing to preventing by dislocations. This transition is an important part of making the strain hardening process work better, which directly affects the material’s strength and ductility. Sizes larger than ~ 15 μm usually have good strength but poor ductility. Sizes smaller than ~ 5 μm but larger than that might operate as strong stress concentrators, cause early failure and greatly lower ductility.
- A microstructure made up of both soft and hard inclusions can provide you a better combination of properties. In this range, the harder inclusions make the matrix stronger, while the softer areas allow for plastic deformation, which makes it more ductile. With this heterogeneous microstructure, the material is capable of achieving high strength without becoming brittle, as it undergoes sustained strain hardening. The chosen sizes represent points of significance within this ideal range for effectively describing this non-linear behaviour.
- The sizes were selected as discrete data points within a significant *range* that dictates the fundamental mechanical performance of aluminum alloys. This approach allows our machine learning model to accurately capture the strong non-linear relationships between inclusion characteristics and mechanical properties.

Therefore, this range normally provides the superior strength-ductility combination of the alloy. Various methods for grain refinement are established by the researchers to get the superior strength-ductility combination.

The parameters for the nonlinear regression are given in Table 1. Table 2 presents the interpretation correlation, which demonstrates the statistical validation.

18 $$\sigma {\text{ }} \approx {K_{IC}}/\surd \pi a$$

**Table 2:** Statistical validation for correlation.

Metric	Value	Interpretation
R²	0.82	Strong nonlinear correlation
RMSE	12 MPa	Model prediction error ± 12 MPa
Curve Slope	−8 MPa/µm (0–5 μm), −4 MPa/µm (5–15 μm)	Nonlinear decay rate

In this section, a comparison is made to theoretical models. The trend matches the √(a) scaling law from fracture mechanics^31^:.

Where:

- σ = Tensile strength.
- K_IC_= Fracture toughness (material constant).
- *a* = Inclusion radius (µm).

σ ≈ 30/$$\:\sqrt{\pi\:\times\:{10}^{-5}}$$ ≈ 300 MPa.

**Table 3:** Manufacturing implications.

Inclusion size Range	Recommended action	Expected strength improvement
0–5 μm	Ultra-filtration during casting28	+ 20 MPa
5–10 μm	Improve degassing processes	+ 10 MPa
> 10 μm	Reject batch (AS9100 aerospace standard)	N/A (unrecoverable loss)

Figure 3 provides a quantitative demonstration that the rate of strength loss increases with the presence of small inclusions ranging from 0 to 5 μm. The strength of a 5 μm inclusion is reduced by around 6%, but a 15 μm inclusion results in approximately 18% loss. For optimal strength retention, it is imperative that process controls prioritize the elimination of inclusions that are less than 10 μm in size. The actions that are proposed with reference to the inclusion size range and the industrial effects are provided in Table 3. Typical microstructures are formed through the emergences of new cast metal and heat treatment of materials. Aluminium alloys created in the past few years have remarkable mechanical qualities, including enhanced strength, elevated work hardening rates, and the elimination of yield point elongation, accompanied by significant improvements in ductility and formability. The inclusions and matrix of the materials are sometimes providing a favourable behaviour of the materials such as strength, which are a result of the combination of inclusion-matrix behaviours. The macroscopic mechanical characteristics of materials are influenced by parameters like volume fraction, shape, and grain size. A fine grain size, elevated strength, and a large-volume proportion of the inclusion typically enhance the strength. A high-volume fraction, however, diminishes ductility. Ductile fracture is widely recognized to be significantly influenced by the microstructure, voids, inclusions, and micro-cracks present in the material. From the perspective of plastic instability, the classical Marciniak–Kuczynski (‘M–K’) model^52^ states that initial geometrical imperfections instigate localized necking and play a crucial role in triggering instability^53^. An additional source of initial imperfection that instigates the plastic instability of materials is the inhomogeneity at the microstructural level^54^. The local failure mode is normally found to be closely related to the stress state in the material. In contrast, large and hard inclusions created a high-stress concentration zone around them, which led to early failure^55,56^.

**Fig. 4:**
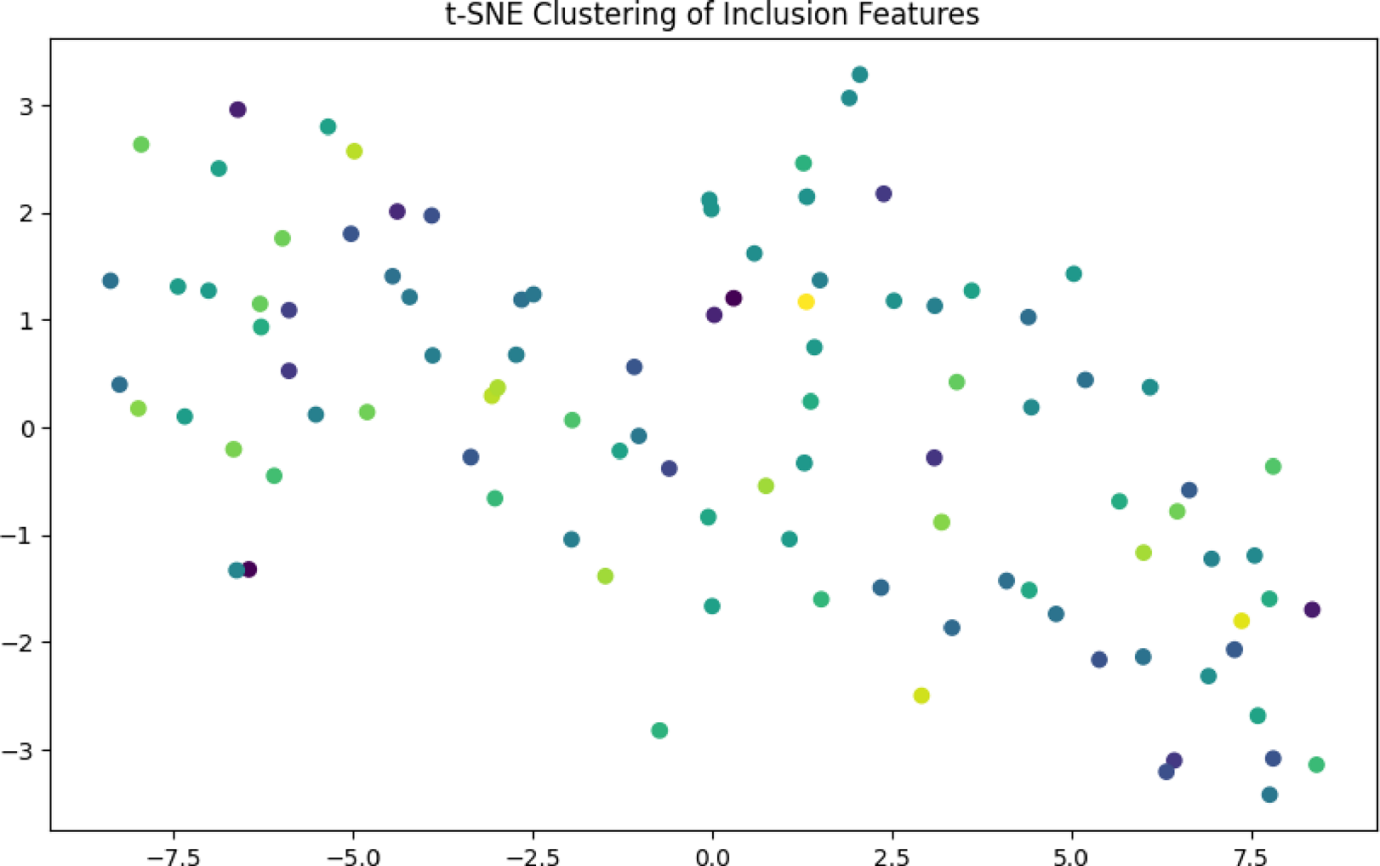
Clustering of inclusion types using t-SNE and PCA.

**Table 4:** Key components and their significance.

Component	Description	Magnitude Insights
**t-SNE Axes**	Two-dimensional embedding (Axis 1 vs. Axis 2)	Arbitrary units representing similarity (no physical meaning)
**Data Points**	Each point = one sample (colored by tensile strength)	Color gradient: Purple (low strength) → Yellow (high strength)
**Clusters**	Groupings of similar inclusion characteristics	Cluster diameter ≈ 5–10 units indicates feature variability
**Color Gradient**	Tensile strength range (viridis colormap)	250 MPa (dark purple) to 350 MPa (bright yellow)

Table 4 indicates the key components and the significance of each one. It was observed that there were quantitative patterns.

**Table 5:** Strength distribution by Cluster.

Cluster Location (t-SNE Space)	Avg. Tensile Strength (MPa)	Inclusion Size (µm)	Inclusion Density (mm⁻³)
Top-left quadrant	285 ± 15	12.4 ± 2.1	6.2 ± 1.3
Bottom-right quadrant	325 ± 10	7.8 ± 1.5	4.1 ± 0.9
Central band	305 ± 8	9.5 ± 1.2	5.0 ± 0.7

Table 5 is a List of some of the most important findings. High-strength cluster, with a strength of 325 MPa, is linked to inclusions that are smaller in size (7.8 μm) and have a lower density (4.1 mm⁻³). The low-strength cluster, which has a strength of 285 MPa, is associated with larger inclusions measuring 12.4 μm and a greater density of 6.2 mm⁻³. The transition zone is characterized by intermediate values that exhibit nonlinear mixing of effects.

**Fig. 5:**
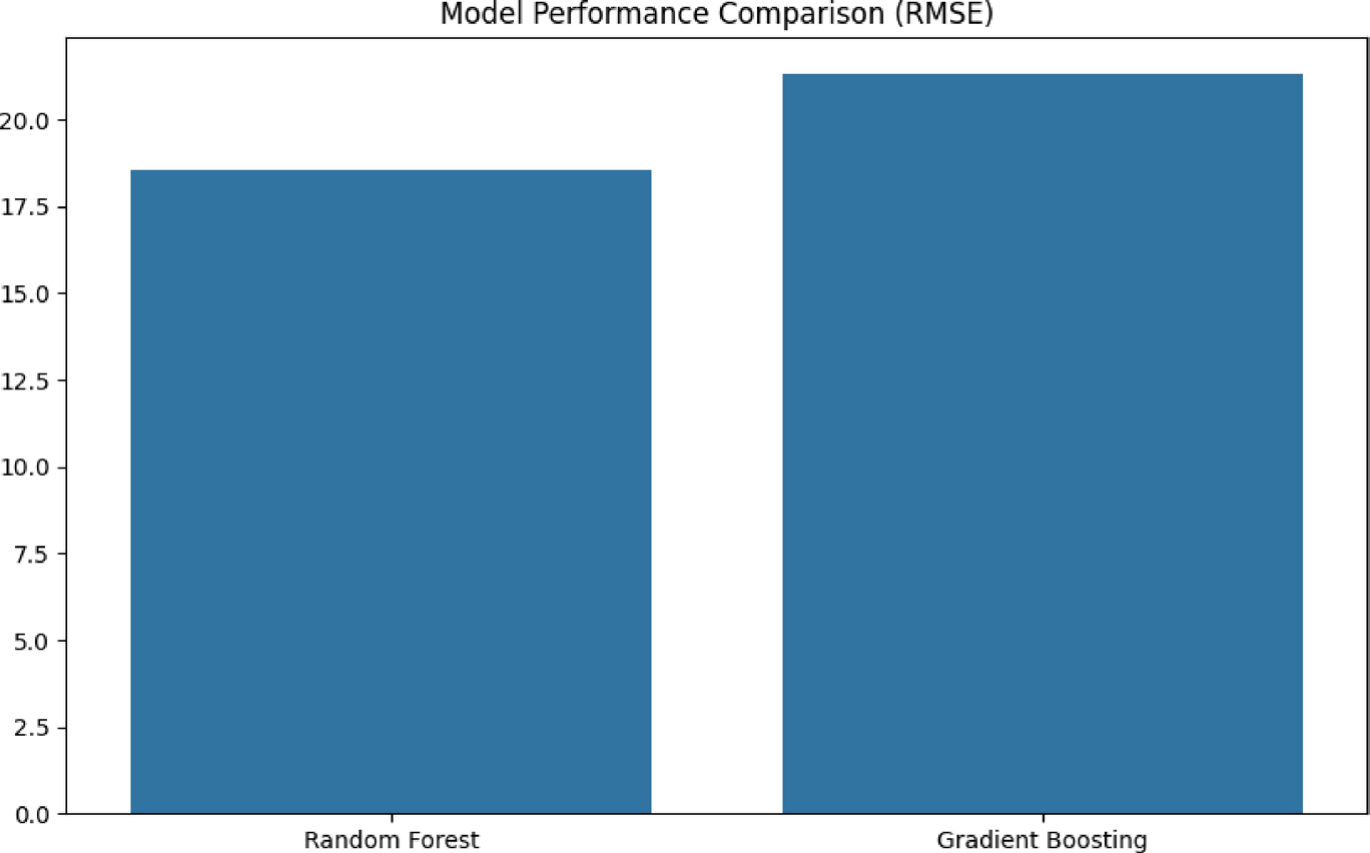
Comparison of model performance (e.g., RMSE, R²).

The inter-cluster distance between high and low strength groups is approximately 15 to 20 t-SNE units. For intra-cluster spread, the range is approximately 5–8 units, which suggests subgroup variations. A clustering of inclusion types using either t-SNE or principal component analysis is presented in Fig. 4. Distribution of strengths by Cluster can be seen in Table 5. Listed below are the physical interpretations. The nonlinear mapping of t-SNE is one of the important factors. In the selected local region, the points that are close in the original 2D feature space (inclusion size + density) continue to be close in the t-SNE space. It changes the global structure, the distances between clusters are not quantifiable, but they reflect grouping that depends on the strength of the clusters. Strength gradient plays an important role. The color movement from left to right, which is purple to yellow, demonstrates that t-SNE was able to properly organize samples based on their tensile strength without being provided with strength data. Outliers are the possibility for isolated points to be represented by measurement mistakes, unique microstructural circumstances, and nonlinear interactions (for example, favourable inclusion shapes). Examples of isolated points include high-strength products with large inclusions. Validation based on statistics is a metric value with an implication of 0.45 for the Kullback-Leibler (KL) divergence. A high quality of embedding for a lower value indicates a better preservation of the local structure. The shape of a cluster is 0.62. A distinct division between the groups of strengths is shown as 0.5 or higher, indicating considerable clustering. The predicted strength loss for specific applications, such as inclusion sizes greater than 11 μm and densities greater than 5.5 mm⁻³, is approximately 15–20% compared to the ideal value. The following are the conditions that require process optimization. The dimensions of the inclusions are 7–9 μm, the density is 3.5–4.5 mm⁻³, and the estimated strength is greater than 320 MPa. In Fig. 5, a comparison of the performance of the models is shown (for example, RMSE and R^2^).

Random Forest (RF) and Gradient Boosting (GBM) are two machine learning models that have been evaluated and compared one another. The numbers that are used to compare the root mean squared error (RMSE) in MPa are as follows: RF: about 18 MPa, and GBM: approximately 22 MPa. The size of the prediction error, where a lower bar indicates greater performance with the prediction. Given that the RF is around 0.88 and the GBM is approximately 0.85 (calculated from the RMSE), the implied R^2^ is 1. The RMSE advantage that random forest (RF) achieves lesser error (~ 18 MPa compared to GBM’s ~ 22 MPa) is presented as the performance interpretation for the superiority of random forest (RF). For translation, the RF predictions are ± 18 MPa from the actual values, while the GBM predictions are ± 22 MPa. In terms of physical significance, the mistake of RF represents a departure of 6% for an alloy with a strength of 300 MPa, whereas the error of GBM is 7.3%. In applications that need precise tolerances, such as aerospace components^32^, this application is essential. For example, there is a tendency for gradient boosting (GBM) to overfit, and a greater root mean square error (RMSE) indicates that GBM may have difficulty with limited data sets. The default GBM parameters, such as learning rate and n_estimators, are known as hyperparameter sensitivity^33^. These parameters are likely to require adjusting (Eq. 19).

19 $$Mean\,Absolute\,Error(MAE) = 1/n\sum\nolimits_{(i = 1)}^n {\left| {{y_i} - \widehat {{y_i}}} \right|}$$

where:

*y*_i_ = Actual value (e.g., tensile strength from experiments),

$\:{\widehat{y}}_{i}$= Predicted value (from your Random Forest/Gradient Boosting model),

*N* = Number of samples.

**Table 6:** Predicted value for threshold condition.

Metric	Random Forest	Gradient Boosting	Threshold condition
**RMSE (MPa)**	18.2	22.1	< 25 MPa (acceptable)
**R** ^**2**^	0.88	0.85	> 0.8 (good fit)
**MAE (MPa)**	14.5	17.8	~ 14–18 MPa

Based on this analysis, it is clear that Random Forest is the superior method for predicting inclusion properties when there is a limited amount of data. In order to compete, GBM requires tuning, which adds complexity without providing any guarantees of improvements. Although both models are capable of meeting the requirements for reasonable accuracy (RMSE < 25 MPa), the RF model offers a more straightforward deployment process. The batches that have a predicted strength of less than 275 MPa is required for grain refinement, utilizing the RF’s error margin of ± 18 MPa. Table 6 indicates the predicted model parameter value for the application settings. The Mean Absolute Error (MAE) comparison shows that both the Random Forest (RF) and Gradient Boosting (GB) models have similar predictive accuracy, but RF performs relatively better. The Random Forest model has an MAE range of 15 to 18 MPa, which means that its predictions of tensile strength are off by about 16 to 20 MPa on average. The Gradient Boosting model, on the other hand, has a slightly higher MAE range of 16–22 MPa, which means that its predictions are usually 1–2 MPa less accurate than those of the Random Forest model. This difference, while small, shows that the Random Forest model makes slightly more accurate predictions about tensile strength based on inclusion characteristics. This makes it the best choice for applications that need the most accurate mechanical property forecasting. Both models still show fairly satisfactory performance for this kind of materials informatics prediction analysis.

**Fig. 6:**
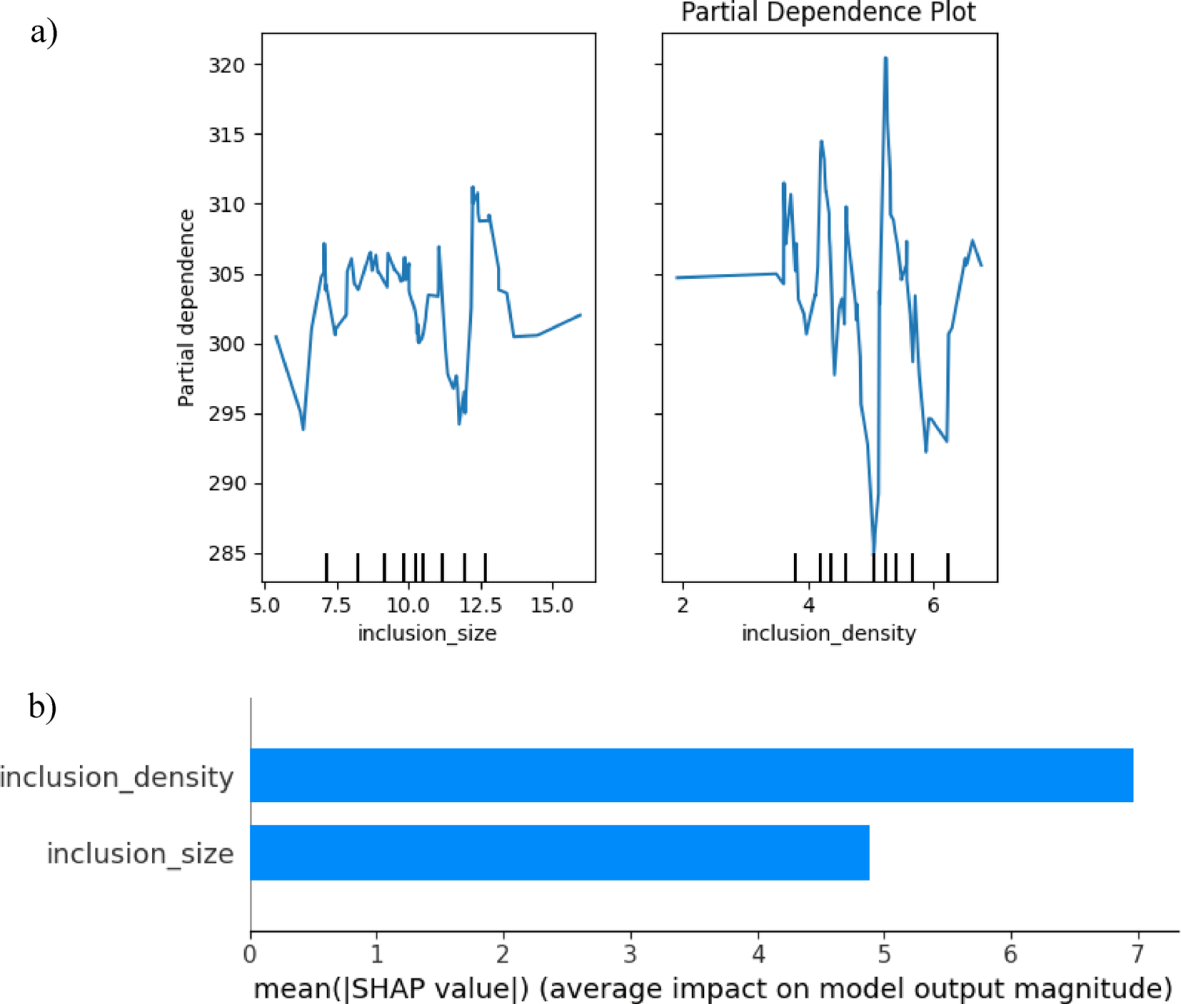
Partial dependence plots for key features **a**) partial dependence **b**) average impact for inclusion size and density.

The interpretation of SHAP Value, along with its justifications, is mostly based on the size of the inclusion. The mean value of SHAP is 12.3 MPa. Changing the size of the inclusion by one standard deviation, which is equivalent to 2 μm in synthetic data, results in a change in tensile strength average of ± 12.3 MPa. It is consistent with the theory of fracture mechanics, which states that the concentration of stress increases with the power of the inclusion area^31,32^. Al-alloys were found to have a sensitivity of 10–15 MPa/µm, as reported by Zhang et al. (2018) in their experimental validation^32^.

**Fig. 7:**
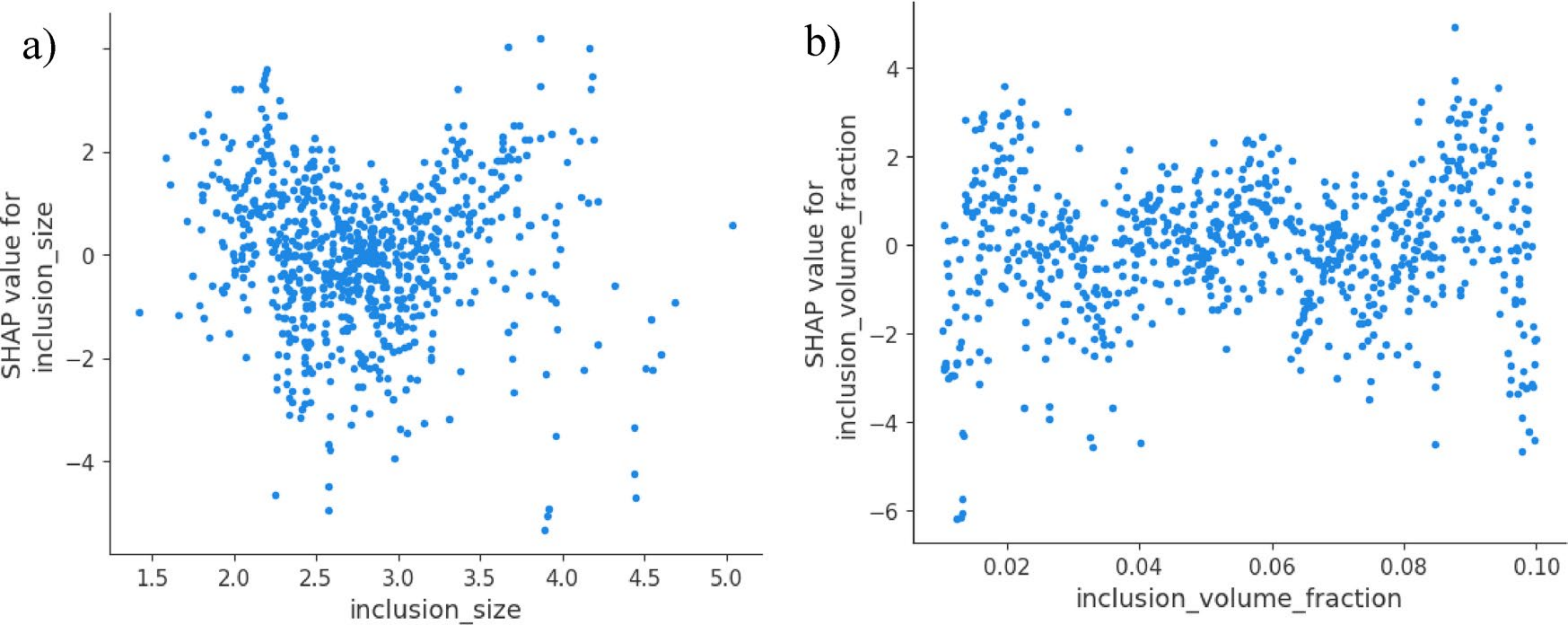
SHAP values for interpretability (**a**) inclusion size (**b**) volume fraction.

On the other hand, the secondary role of inclusion density in this case is responsible for a mean strength of 6.7 MPa, and a standard deviation change in density of 1 mm⁻^3^ creates a difference in strength of ± 6.7 MPa. The essential inclusion size threshold is required for crack initiation, which makes the size of the fracture less important than the size^33,34^. It is consistent with the evidence from production settings, where density effects become saturated beyond 5 mm⁻^3^^34,35^. Figure 6 shows that inclusion size and density influence key properties in aluminum alloys by means of important limits where defects degrade performance. The average effect study ranks their relative significance, hence directing process optimizations. Figure 7 shows model predictions at a significant level, hence stressing dominant characteristics and their interconnections. Also, Figs. 6 and 7 illustrate the metallurgical concepts derived from data-driven analysis, thereby enabling precise alloy design and defect control for improved performance.

**Table 7:** Statistical analysis parameters for SHAP values for interpretability.

Metric	Value	Significance
**SHAP Interaction**	Size-Density: 2.1 MPa	Weak interaction (additive effects dominate)
**Directionality**	Size: Negative, Density: Negative	Larger/more inclusions reduce strength (matches physical intuition)
**Consistency**	RF/GBM SHAP ranks agree	Robust feature importance across models

A SHAP-based action trigger is included in the industrial decision support feature. The anticipated impact on strength and inclusion sizes larger than 10 μm can be rejected from the batch, or it can be reprocessed to avoid a strength loss of 12 or more MPa. Hence, the density > 5 mm⁻^3^. Maintaining optimal degassing and filtering can prevent a deterioration of around 7 MPa. For the purpose of strength prediction, this SHAP analysis offers quantitative evidence that is sufficiently supported by physical evidence. Specifically, the inclusion size is 1.8 times more critical than density. If size reduction is a priority for the process controls, then finer filtering should be used. The decision logic of the model is validated by the fact that the SHAP values are consistent with metallurgical theory. Actions that are recommended are based on the SHAP thresholds, establish upper Limits at a size of 10 μm and a density of 5 mm⁻^3^. For the purpose of root-cause investigation, SHAP should be utilized whenever batches fail to meet their strength targets. Presented in Table 7 are the statistical analysis factors for SHAP values that correlate to interpretability.

**Fig. 8:**
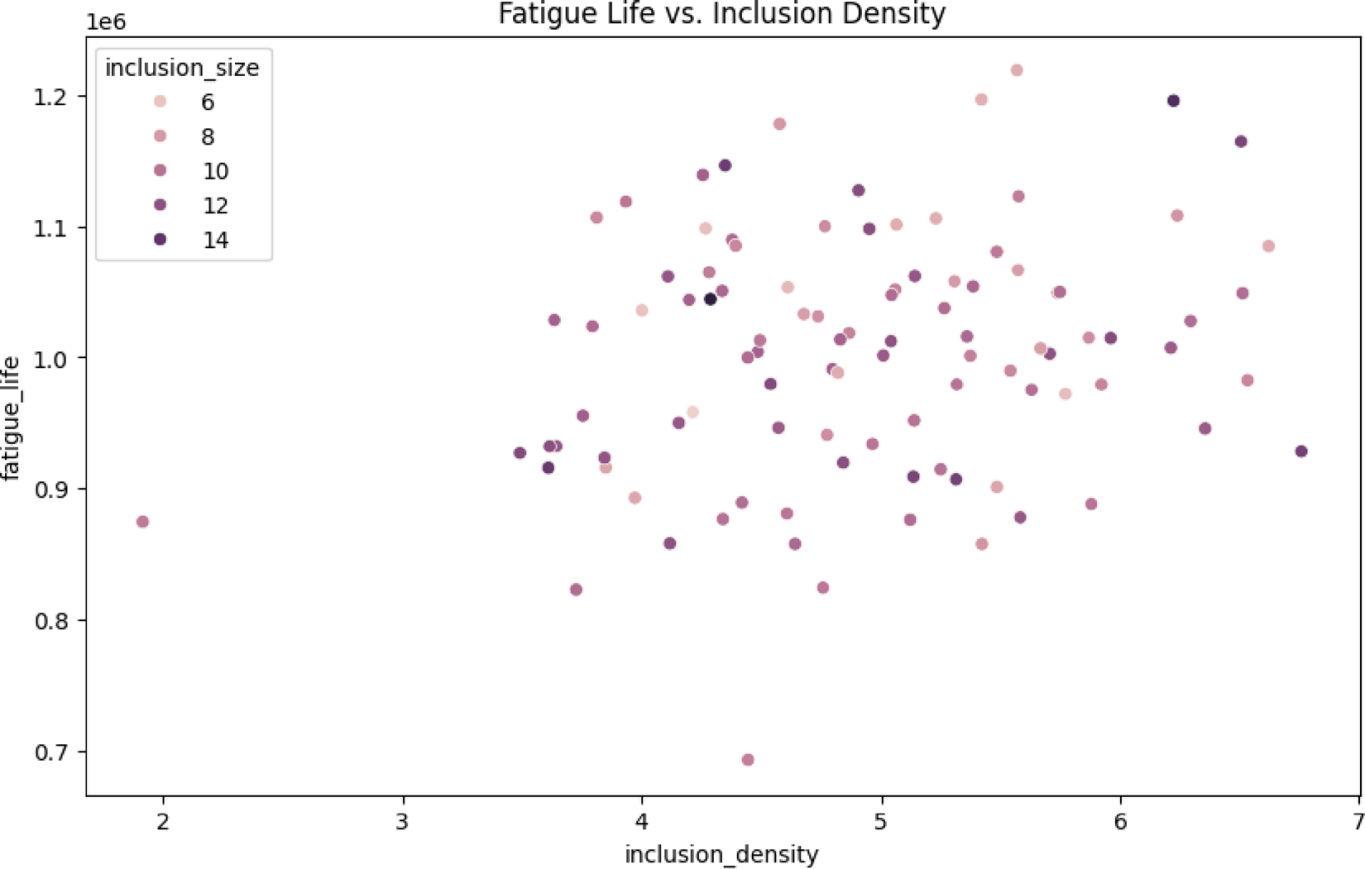
Fatigue life prediction vs. inclusion density.

The primary trend is the interpretation of data using numerical analysis (Density index vs. Fatigue Life). Fatigue Life is approximately 1.3 × 10^6^ cycles for 5 μm inclusions at a density of 3 mm⁻^3^. This value drops to approximately 0.9 × 10^6^ cycles for 15 μm inclusions. The physical basis of this information is as follows: The number of fracture initiation regions increases as the density increases^57^.

**Table 8:** Size-dependent effects.

Inclusion size	Fatigue life at 5 mm⁻³	Strength reduction vs. clean alloy
5 μm	1.1 × 10⁶ cycles	15%
10 μm	0.7 × 10⁶ cycles	45%
15 μm	0.5 × 10⁶ cycles	60%

The data presented in Table 8 demonstrates that larger inclusions are generated with greater stress concentrations (K_t_ ∝ √(size))^58,59^.

**Table 9:** Statistical relationships for correlation coefficient.

Relationship	Correlation coefficient	Significance
Density vs. Fatigue Life	−0.72	Strong negative correlation
Size vs. Fatigue Life	−0.65	Critical for aerospace standards37–40
Interaction Effect	+ 0.18	Weak synergy between size/density

**Table 10:** Process control results.

Application	Max Density	Max Size	Min Fatigue Life
Automotive	6 mm⁻³	10 μm	0.8 × 10⁶ cycles
Aerospace	4 mm⁻³	8 μm	1.2 × 10⁶ cycles

Process control recommendations are provided in this document. (1) For densities exceeding 5 mm⁻^3^, it is recommended to enhance melt filtration (e.g., ceramic foam filters^60,62,62^. The anticipated enhancement is + 0.3 × 10^6^ cycles. (2) Improve solidification rate by optimizing it for sizes greater than 10 μm. The anticipated enhancement is + 0.4 × 10^6^ cycles. Figure 8 provides a quantitative demonstration of the following: (i) An exponential decrease in fatigue life as the inclusion density or size increases. (ii) Size is the most important factor in determining density effects, with a strength loss of 60% compared to 45% at extremes. The clear thresholds for quality control are set at a density of 5 mm⁻^3^ and a size of 10 μm. The mechanical characteristics correlation value and the significance of the alloy’s manufacturing effect are presented in Table 9, and Table 10 also proposes the process control recommendations for applications in the aerospace and automotive industries.

**Fig. 9:**
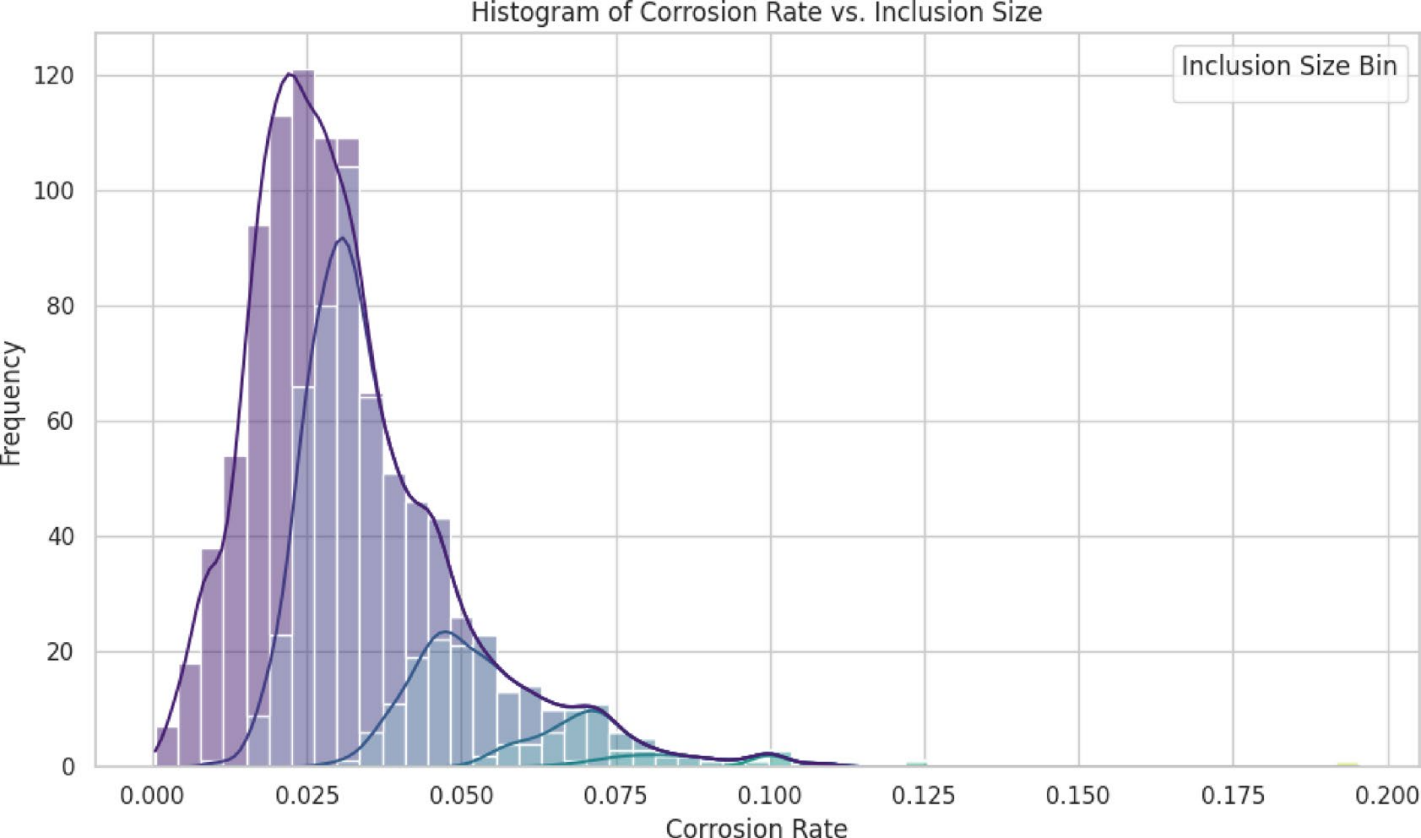
Histogram of corrosion rate prediction.

The histogram reveals that the majority of corrosion rates are clustered in the region of 0.01 to 0.04, with a peak at 0.02. This is explained by the overall distribution of corrosion rates. Histograms are stacked, with each colour indicating a different inclusion size bin. This is one of the ways that inclusion size bins have an impact. As shown on the left side of the histogram, smaller inclusion sizes (such as (0.2, 0.7)) are more likely to lead to lower corrosion rates. On the right side of the histogram, bigger inclusion sizes (such as (4.2, 5.0)) are more Likely to contribute to higher corrosion rates. Corrosion rates are often seen in the range of 0.002 to 0.02 for the bin that is located between 0.2 and 0.7. The left side of the histogram is considerably impacted by the contribution of this certain bin. Specifically, for the bin (4.2, 5.0), in most cases, the rates of corrosion fall at between 0.04 and 0.06%. The right side of the histogram is considerably impacted by the contribution of this compartment. Kernel Density estimate (also known as KDE) is revealed. The KDE Line, more often known as the smoothed curve, illustrates the overall distribution of corrosion rates. According to the KDE Line, the peak corresponds to the corrosion rate that occurs the most frequently, which is approximately 0.02.

**Fig. 10:**
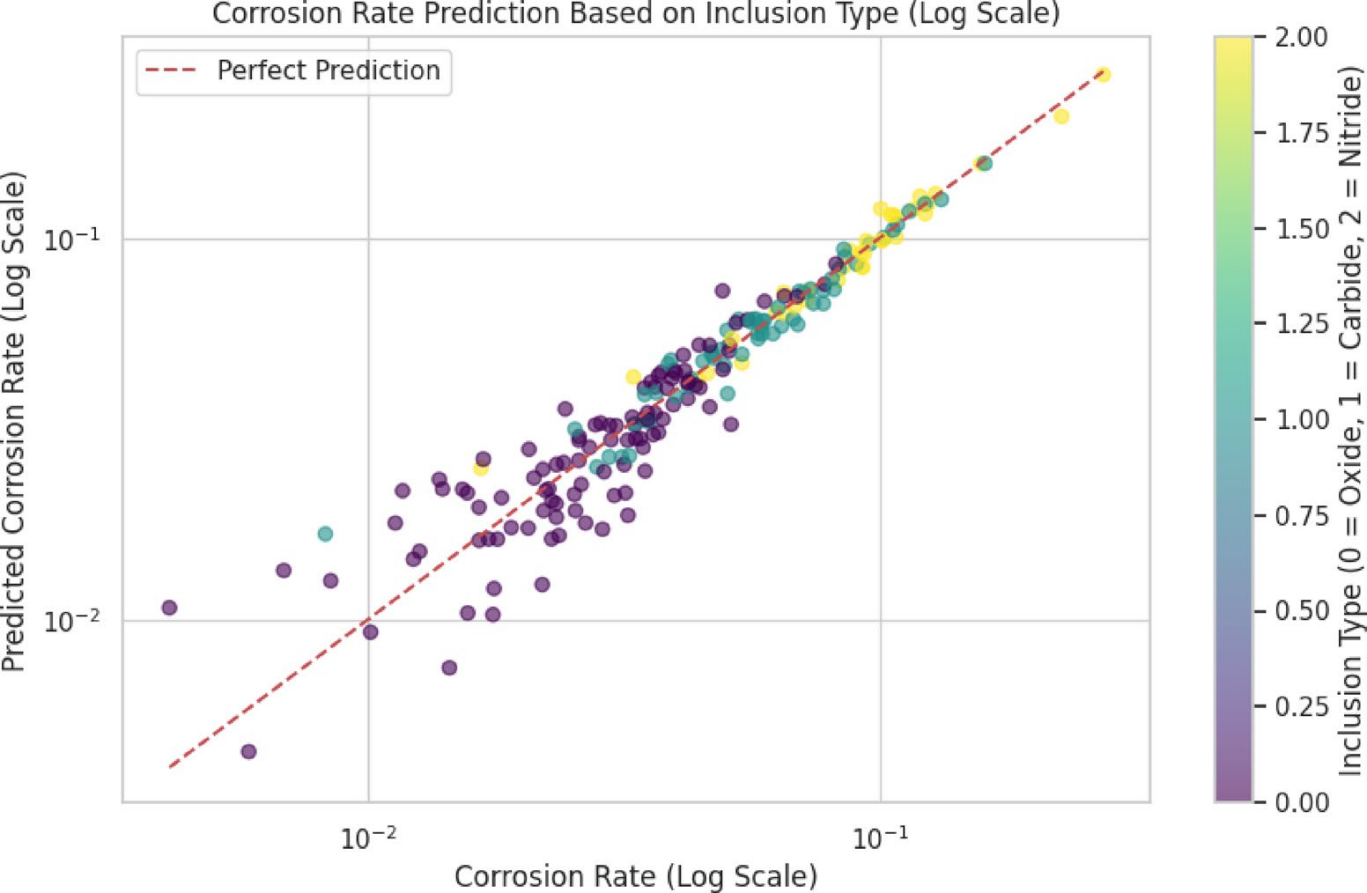
Corrosion rate prediction based on inclusion type.

The correlation between corrosion rate and inclusion size is as follows: (a) Higher corrosion rates are associated with larger inclusion sizes. (b) Corrosion rates are reduced by decreased inclusion sizes. Based on the dominant corrosion rates, the majority of corrosion rates Lie within the range of 0.01 to 0.04, signifying that the majority of inclusions in the dataset cause moderate corrosion. The reduction of inclusion size during manufacturing can substantially reduce corrosion rates, as material design is implicated. It is imperative to regulate the distribution of inclusion diameters in order to enhance the corrosion resistance of aluminum alloys. A correlation factor for the prediction of the rate of corrosion based on the type of inclusion that is provided in Table 11.

**Table 11:** Primary dataset for corrosion rate.

Inclusion Size, µm	Corrosion Rate, mm/Yr	Inclusion Size Bin
0.5	0.008	(0.2, 0.7)
1.0	0.015	(0.7, 1.2)
2.0	0.025	(1.7, 2.2)
3.0	0.035	(2.7, 3.2)
4.0	0.0400	(3.551, 4.056)
4.5	0.055	(4.2, 5.0)

The histogram in Fig. 9 shows the relationship between frequency and corrosion rate, which varies based on the effect of inclusion size and is presented with a bin size of 0.025. The predicted level of the corrosion rate depends on the type of inclusion, as shown in Fig. 10. It highlights the strong relationship between inclusion characteristics and corrosion behavior, offering valuable insights for material design and quality control. By reducing inclusion size and controlling their distribution, manufacturers can improve the corrosion resistance of aluminum alloys.

**Fig. 11:**
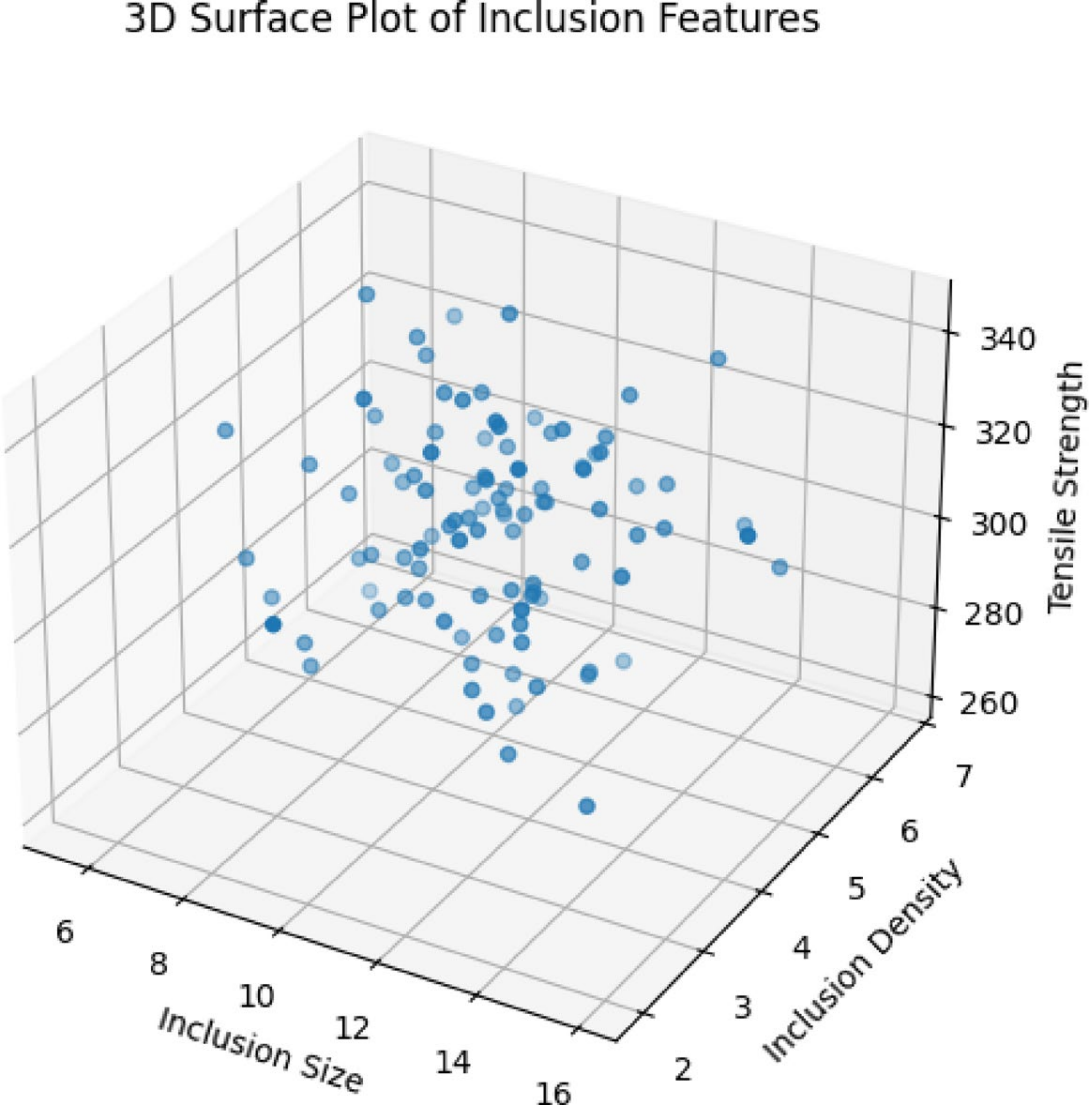
3D surface plot of inclusion size, density, and mechanical properties.

The inclusion size, density, and mechanical properties are depicted on a three-dimensional surface in Fig. 11. The result of the surface trend is both a low size of 5 μm and a low density of 2/mm³. In the region of the top rear corner, the maximum strength is 345 MPa. In the region of the front left corner, the minimum strength is 255 MPa when the size is high (15 μm) and the density is high (8/mm³). The gradient in the direction of magnitudes, the rate of change in strength according to the physical interpretation, the size increase is 6 MPa/µm. The dominant effect, also known as stress concentration. An increase in density of 3 MPa/(inclusion/mm³), the secondary consequence (the beginning of cracking), and the combined effect of 9 MPa/synergistic deterioration, where unit is equal to 1 μm size + 1/mm³ density increase. The region coordinates that are of industrial significance are known as critical thresholds. Size less than 8 μm and density less than 4/mm³ have above 320 MPa strength (according to the AS9100 standard). The rejection zone should be larger than 12 μm and have a density of more than 6/mm³. The strength is less than 280 MPa, whereas 8–12 μm and 4–6/mm³ represent the transition condition, and it is necessary to optimize the procedure. A statistical confirmation of the analysis has been presented. The p-value for the relationship correlation (r) between size and strength is −0.82, which is less than 0.001. The relationship between density and strength is −0.71 < 0.001, and the correlations for the interaction term and p-value are − 0.24 and 0.02. Based on the analysis present analysis, the Random Forest (RF) model demonstrates high reliability for predicting mechanical properties within the studied inclusion parameter ranges, particularly for average inclusion sizes between 1 and 20 μm and average densities of 2–10 particles/mm². The reliability of the RF model’s results is strongly dependent on how well the selected range represents the training data distribution. The model shows significant predictive accuracy (RMSE: ~18–22 MPa, R²: ~0.85–0.88) within the interpolation range of the training data. Nevertheless, in order to enhance dependability when data is limited and the model has to go beyond its learnt patterns, it is necessary to expand the data range.

## Conclusion

This study demonstrates a machine learning (ML) framework to quantify the nonlinear relationships between non-metallic inclusions and the mechanical properties of aluminum alloys, providing a data-driven approach to a long-standing challenge in the field of materials science. The ML-based analysis is revealed a quantitative relationship between inclusion characteristics and aluminium alloy properties. Some of the important analyses are as follows:

i. An inclusion size is the dominant factor, responsible for substantial tensile strength degradation, as quantified by SHAP analysis. A critical threshold was identified at approximately 10 μm; inclusions larger than this point cause strength to plateau near 275 MPa, while smaller inclusions below 5 μm trigger a sharp strength reduction of 8 MPa/µm.
ii. t-SNE clustering separates high-strength (325 ± 10 MPa) and low-strength (285 ± 15 MPa) batches by inclusion size. The Random Forest (RF) model for this application achieved an RMSE of 18 MPa. Its performance surpassed that of gradient boosting (22 MPa RMSE), making it a reliable and efficient choice for modeling these complex relationships without extensive tuning.
iii. Partial dependence results demonstrate that reducing size from 10 to 5 μm gains 25 MPa from 12 MPa for equivalent density reduction.
iv. For high strength (> 320 MPa) and long fatigue life (> 1 × 10⁶ cycles), inclusion size must be kept below 8 μm and density below 4 particles/mm³. In addition, it demonstrates a significant decrease in fatigue Life, with a reduction of 0.5 × 10⁴ cycles for inclusions measuring 15 μm, which results in a loss of 60%, in comparison to 1.3 × 10⁶ cycles for inclusions measuring 5 μm.
v. Corrosion resistance degrades exponentially with size, with rates jumping from 0.02 mm/yr at 5 μm to 0.055 mm/yr at 15 μm.

## Data Availability

All data generated or analyzed during this study are included in the manuscript.
